# Over-expression of a NAC 67 transcription factor from finger millet (*Eleusine coracana* L.) confers tolerance against salinity and drought stress in rice

**DOI:** 10.1186/s12896-016-0261-1

**Published:** 2016-05-11

**Authors:** Hifzur Rahman, Valarmathi Ramanathan, Jagedeeshselvam Nallathambi, Sudhakar Duraialagaraja, Raveendran Muthurajan

**Affiliations:** Department of Plant Biotechnology, Centre for Plant Molecular Biology and Biotechnology, Tamil Nadu Agricultural University, Coimbatore, 641 003 India

**Keywords:** Transcription factor, Abiotic stress, Genetic engineering, Drought, Salinity

## Abstract

**Background:**

NAC proteins (NAM (No apical meristem), ATAF (Arabidopsis transcription activation factor) and CUC (cup-shaped cotyledon)) are plant-specific transcription factors reported to be involved in regulating growth, development and stress responses. Salinity responsive transcriptome profiling in a set of contrasting finger millet genotypes through RNA-sequencing resulted in the identification of a NAC homolog (*EcNAC* 67) exhibiting differential salinity responsive expression pattern.

**Methods:**

Full length cDNA of *EcNAC67* was isolated, characterized and validated for its role in abiotic stress tolerance through agrobacterium mediated genetic transformation in a rice cultivar ASD16.

**Results:**

Bioinformatics analysis of putative NAC transcription factor (TF) isolated from a salinity tolerant finger millet showed its genetic relatedness to NAC67 family TFs in related cereals. Putative transgenic lines of rice over-expressing *EcNAC*67 were generated through *Agrobacterium* mediated transformation and presence/integration of transgene was confirmed through PCR and southern hybridization analysis. Transgenic rice plants harboring *EcNAC67* showed enhanced tolerance against drought and salinity under greenhouse conditions. Transgenic rice plants were found to possess higher root and shoot biomass during stress and showed better revival ability upon relief from salinity stress. Upon drought stress, transgenic lines were found to maintain higher relative water content and lesser reduction in grain yield when compared to non-transgenic ASD16 plants. Drought induced spikelet sterility was found to be much lower in the transgenic lines than the non-transgenic ASD16.

**Conclusion:**

Results revealed the significant role of *EcNAC67* in modulating responses against dehydration stress in rice. No detectable abnormalities in the phenotypic traits were observed in the transgenic plants under normal growth conditions. Results indicate that *EcNAC67* can be used as a novel source for engineering tolerance against drought and salinity stress in rice and other crop plants.

**Electronic supplementary material:**

The online version of this article (doi:10.1186/s12896-016-0261-1) contains supplementary material, which is available to authorized users.

## Background

One of the major challenges in future agriculture is to sustain food grain production under changing climate and limited natural resources such as water and nutrients. Abiotic stresses *viz.*, drought, salinity, flooding and temperature extremes are becoming major threats to increased agricultural productivity under fragile environments. Among these, drought and salinity remain at the top in affecting productivity of major food grains such as rice, wheat, maize etc., and predicted climate change may cause serious salinization of more than 50 % of arable lands by 2050 [1]. This necessitates genetic manipulation of tolerance against these two major abiotic stresses in major food crops which remains very difficult through conventional breeding methods due to complexity of tolerance mechanisms [2, 3]. In this context, advancements in the fields of molecular breeding and genetic engineering offer us a powerful tool for genetic manipulation of these traits [3]. During the recent past, genetic engineering has been successful in developing transgenic crop plants engineered for their tolerance against biotic/abiotic stresses, enhanced nutritional quality and various agronomic traits [4–6]. Genetic engineering strategies for improving salinity tolerance in crop plants includes manipulation of various metabolic pathways *viz.*, accumulation of osmolytes, antioxidant enzymes, regulating the uptake/compartmentalization of salts, transcriptional factors and various signalling pathway components etc., [7]. In this context, identification and validation of novel genes associated with various component traits controlling salinity tolerance in candidate crops/organisms is an important step which will allow us to manipulate salinity tolerance in any agriculturally important crop.

Recently, numerous studies have shown that transcription factors (TFs) play an important role in regulating responses against various stresses in plants and some of them have been shown to be essential for stress tolerance [8]. Genetic manipulation of transcription factors have been demonstrated to be an effective strategy in manipulating complex traits like drought/salinity tolerance rather than modification of individual genes involved in key metabolic pathways due to the ability of TFs in modulating expression of hundreds of downstream genes involved in various metabolic pathways associated with stress tolerance in plants [8–11]. Several studies have clearly demonstrated that numerous TFs, such as DREB, bZIP, zinc-finger, MYB, WRKY and NAC, directly or indirectly regulate plant responses under abiotic stress conditions [11–14]. The NAC protein (NAM, No apical meristem; ATAF, Arabidopsis transcription activation factor and CUC, Cup-shaped cotyledon) super-family is one of the largest plant-specific TF families containing a highly conserved N-terminal DNA-binding domain, a nuclear localization signal sequence and a variable C-terminal domain [15–18]. Around 138, 158, 149 and 289 NAC family members have been reported from *Arabidopsis*, Rice, *Setaria* and *Populus trichocarpa,* respectively [19]. NAC TFs play an important role in growth, development including pattern formation of embryos and flowers [20], secondary wall formation [21, 22], leaf senescence [23] and root development [15] in plants. Besides being involved in plant growth and development; NAC TFs were reported to be involved in modulation of responses against various biotic and abiotic stresses [10, 24–26] showing their potential to improve biotic and abiotic stress tolerance through genetic engineering [27].

Several genomics and bioinformatics studies have led to identification of number of drought/salinity responsive NAC TFs in Arabidopsis [28, 29], rice [30] and soy bean [31]. Results of above studies suggest that stress-responsive NAC TFs may have important roles in providing tolerance against abiotic stresses and their over-expression can improve stress tolerance in crop plants. Transgenic rice plants engineered with a NAC TF (*OsNAC6*) were found to exhibit enhanced tolerance against various abiotic (drought and salinity) and biotic stresses [32]. Similarly, transgenic cotton plants engineered with a stress responsive NAC TF namely *SNAC1* in rice were found to exhibit enhanced tolerance against drought and salinity stresses. The transgenic cotton plants were found to possess enhanced root development and reduced transpiration rate [33]. Similar observations were reported when rice plants were engineered with *OsNAC10* [34] and *SNAC1* [18]. In another study, *TaNAC67* from wheat was found to improve tolerance against drought, salinity and freezing stresses in *Arabidopsis* [35].

It has been hypothesized that exploitation of highly saline tolerant “halophytes” or wild germplasm may serve as an excellent strategy for understanding physiological/molecular mechanisms underlying salinity tolerance, and thereby, leading to identification of novel candidate gene(s) for engineering salinity tolerance in agriculturally important crop plants [36–38]. Finger millet (*Eleusine coracana* L.) is one of the resilient cereal crops belonging to the family, Poaceae and genetically close to rice [39] which is known for its high degree of tolerance against drought, salinity and blast disease [40, 41]. Our earlier studies on transcriptome profiling of salinity responsiveness in a set of contrasting finger millet genotypes resulted in the identification of several novel putative candidate genes for functional validation [42]. In the present study, efforts were made to isolate and validate the function of a novel NAC transcription factor namely *EcNAC67* exhibiting contrasting salinity responsive expression pattern between the susceptible and tolerant finger millet genotypes. Full length gene encoding NAC transcription factor *i.e. EcNAC67* was isolated from a salinity tolerant finger millet genotype, Trichy 1, and transgenic plants of a rice variety ASD16 over-expressing *EcNAC67* were developed and evaluated for their responses against drought and salinity stresses.

## Methods

### Genetic material and stress treatments

Based on the results of our earlier study [42], a putative candidate gene namely a NAC transcription factor 67 (*EcNAC67*) exhibiting contrasting salinity response between susceptible (CO 12) and tolerant (Trichy 1) finger millet genotypes was selected for functional validation. Saline tolerant finger millet genotype “Trichy 1” was grown under normal greenhouse conditions up to 21 days (when plants were 4–5 leaf stage) and salinity stress was imposed by adding 300 mM of NaCl by maintaining suitable control plants irrigated with normal water. Leaf (top 3 leaves), root and shoot tissues were collected from both control and salinity stressed plants (20 days after stress) of Trichy 1 and used for expression analysis.

### Expression analysis of *EcNAC67*

Tissue samples collected from control and salinity stressed plants of Trichy 1 were frozen in liquid nitrogen and used for total RNA extraction as per manufacturer’s protocol (Biobasic Inc., Canada). Equal amount of DNAse treated total RNA (about 1 μg) was converted to sscDNA using Transcriptor High Fidelity cDNA Synthesis Kit (Roche, Germany) and used for qRT-PCR analysis (StepOne Plus, Applied Biosystems, USA) by following default cycling conditions (10 min 95 °C, 40 cycles of 15 s at 95 °C and 60 s at 60 °C). The reaction mixture contained SYBRGreen Master mix (Roche Diagnostics) 300 nM of gene specific primers (NAC RT-F 5’-TCAGCAGCAGATGATGGTG-3’ and NAC RT-R 5’-CGGATCAGGTTCAGGTTCTTCG-3’) (see Additional file 1) and 2 μl of cDNA in each 15 μl reaction. “No template controls” (NTC) containing all of the RT-PCR reagents except the cDNA template were also maintained to rule out cross contamination. Abundance (relative quantity) of mRNAs was calculated using the comparative Ct (ΔΔCt method; [43]). qRT-PCR analysis was repeated using samples collected from three biological replications including two technical replications per biological replication and Actin was used as an endogenous reference gene for the normalization of Ct values (see Additional file 1).

### Isolation and characterization of cDNA encoding *EcNAC67*

Gene specific primers were designed for isolating full length cDNA encoding for the candidate NAC transcription factor (*EcNAC67)* based on the alignment of finger millet transcript reads against its rice homologue at both 5’ and 3’ UTRs [42]. Primers were designed with the flanking restriction sites viz., *Bam*HI in the forward primer (5’-cgc *ggatcc* CAG GAG GGA GAG AGG AAA GAG-3’) and *Kpn*I site in the reverse primer (5’- cgc*ggtacc* C GGA TCA GGT TCA GGT TCT TCG-3’). Full length cDNA encoding *EcNAC67* was PCR amplified (150 ng of each primer, 200 mM dNTPs, 2.5 U XT5 DNA polymerase in a 50 mL reaction, with 94 °C, 5 min for 1 cycle, 94 °C, 1 min, 60 °C, 1 min, and 72 °C, 1.5 min for 30 cycles; and 72 °C, 7 min for 1 cycle) from the sscDNA synthesized from salinity tolerant finger millet genotype Trichy1. Amplified PCR products were purified and cloned in pTZ57RT vector as per manufacturer’s protocol using InsTAclone PCR Cloning Kit (ThermoScientific, USA) and sequenced (SciGenom Labs, India).

### *In silico* analysis

Nucleotide and translated amino acid sequence analysis was performed using BLASTn/BLASTp search against RNA/cDNA/protein sequences in the NCBI database (http://blast.ncbi.nlm.nih.gov/Blast.cgi). Multiple sequence alignment of deduced amino acid sequences of *EcNAC67* against other known NAC sequences from related crop species was carried out using CLUSTALW tool in BioEdit software and used for phylogenetic analysis (MEGA6 software using the maximum likelihood method with 1,000 bootstrap replications). Various other properties viz., secondary structure (PSIPHRED; http://bioinf.cs.ucl.ac.uk/psipred/); pI/Mw (Compute pI/Mw; http://web.expasy.org/compute_pi/); functional region (PROSITE; http://www.expasy.org/) and subcellular localization (ProtCompv9.0; http://www.softberry.com/berry.phtml?topic = protcomppl) were also analyzed.

## Functional validation of *EcNAC*67

### Construction of plant transformation vector harboring *EcNAC67*

Full length cDNA encoding *EcNAC67* was released from pTZ57RT through *Bam*HI/*Kpn*I restriction digestion and ligated in a plant transformation vector pCAMBIA1300 under the control of *RD29* promoter and *nos* terminator. Putative recombinant clones were selected and used for confirmation of the presence and orientation of the transgene through PCR analysis (using M13F and M13R primers) and sequencing. Then, pCAMBIA1300 harboring the transgene was mobilized into the *Agrobacterium* strain LBA4404 through freeze thaw method [44].

### Genetic transformation of rice variety ASD16 using pCAMBIA1300 harboring *EcNAC67*

Immature embryos of rice variety ASD16 were co-cultivated with *Agrobacterium* strain LBA4404 harboring pCAMBIA1300 + *EcNAC67* as suggested by Hiei and Komari [45]. Transformed calli were subjected to selection in a medium containing 50 mg/l hygromycin and putative transgenic calli were regenerated in the presence of 1 mg/ml of NAA and 3 mg/ml 6-BA and rooted on half MS media containing 50 mg/lhygromycin [45]. Putative transgenic plants were transferred to transgenic green house for hardening and establishment.

### Molecular characterization of putative transgenic plants (T_0_)

Putative transgenic plants of ASD16 engineered with *EcNAC67* were subjected to PCR analysis for confirming the presence of selectable marker gene (hygromycin), transgene (*EcNAC67*) and *vir* gene using gene specific primers (Additional file 1) and southern hybridization analysis. Genomic DNA isolated from the putative transgenic plants was digested with *Bam*HI, electrophoresed on 1 % agarose gel and blotted onto a positively charged nylon membrane along with suitable non-transgenic ASD16 and hybridized using P^32^ labeled fragments of hygromycin (*hpt*) marker gene [46]. After hybridization, membranes were washed, dried and exposed to X-ray film (Kodak Photo Film) overnight and developed.

### Evaluation of transgenic rice plants engineered with *EcNAC*67 against salinity and drought

Transgenic ASD16 rice plants (T_0_) confirmed through PCR and southern hybridization analysis were selfed and forwarded to T_1_ generation. About 50 plants (T_1_) from each event were raised and subjected to PCR analysis to identify transgene positive and negative plants. Positive transgenic plants in all the events were allowed to grow till maturity in soil filled pots and observations on morphological characters were recorded. Seeds were collected from each plant individually and used for evaluation of tolerance against salinity and drought. Transgenic lines (T_2_) were evaluated for their level of salinity tolerance at both germination and vegetative stage along with non-transgenic (NT) controls. At germination stage, seeds of both transgenic and non-transgenic ASD16 were germinated in petri plates containing different concentrations of NaCl (0 mM, 75 mM, 100 mM and 150 mM). After every 24 h, fresh NaCl solutions of respective concentration were used for replacing existing solutions. Germination percentage, root length (cm) and shoot length (cm) were recorded on 10^th^ day. For vegetative stage screening, seeds of both transgenic and non-transgenic ASD16 were germinated in petri plates (upto 7 days) and then transferred to hydroponics system in trays filled with Yoshida solution (grown up to 30 days). Presence of transgene was again confirmed through PCR analysis and salinity stress was imposed by adding 100 mM NaCl to the Yoshida solution. Effect of salinity stress on both transgenic and non-transgenic plants was assessed based on the development of wilting and drying of leaves. After 35 days of 100 mM NaCl stress, plants were transferred back to normal condition (in pots filled with soil) and allowed to grow till maturity to assess the recovery ability.

Transgenic plants were also evaluated for their tolerance against drought stress. Seeds of different transgenic lines were germinated in por trays and PCR analysis was carried out to identify transgene positive plants. Positive plants were transplanted in pots containing 2.5 kg of soil and kept in trays filled with water along with non-transgenic controls. All the plants were maintained under greenhouse conditions (70 % humidity, 28 °C, 12 h light/12 h dark cycle). Drought stress was imposed by withholding water at 54 days after sowing (DAS) and pots were saturated with water for overnight before imposing the stress. Observations on soil moisture content(%) and leaf relative water content (%) were recorded at 8 days after stress when non-transgenic ASD16 started showing severe stress symptoms. RWC was calculated by using the formula,

$$ RWC\kern0.5em \left(\%\right)\kern0.5em =\kern0.5em \frac{\left(FW\kern0.5em -\kern0.5em DW\right)}{\left(TW\kern0.5em -\kern0.5em DW\right)}\kern0.5em \times 100 $$

Where, FW is fresh weight of leaf, DW is dry weight of leaf and TW is turgid weight of leaf.

### Abiotic stress responsiveness of EcNAC67 in rice

Leaf samples were collected from control and drought/salinity stressed plants of all transgenic lines along with non-transgenic ASD16 plants and used for analyzing the expression of transgene through qRT-PCR as described in previous section (Expression analysis of *EcNAC67*). Salinity responsive expression pattern of all the transgenic lines was compared against non-transgenic ASD16 lines. Ubiquitin was used as an endogenous reference gene for the normalization of Ct values (see Additional file 1).

## Results

### Isolation of a salinity responsive NAC transcription factor from finger millet

In our earlier study [42], RNA-seq analysis was carried out in a set of contrasting finger millet genotypes to monitor the salinity responsive changes at transcript level. A putative candidate NAC domain-containing protein homologous to a rice NAC67 protein (LOC_Os03g60080) was found to be significantly up-regulated in the tolerant finger millet genotype Trichy 1 when compared to the susceptible genotype CO 12. Based on the sequence information of finger millet contigs mapped at 5’ and 3’ UTRs of OsNAC67, gene specific primers were designed to isolate full length cDNA of *EcNAC67* from Trichy 1 genotype. Cloning and sequencing data revealed that *EcNAC67 *was found to be 1178 bp in size including a 36 bp 5’UTR, 969 bp open reading frame and 173 bp 3’UTR.

### Validation of differential regulation of *EcNAC67* during salinity stress

**Fig. 1:**
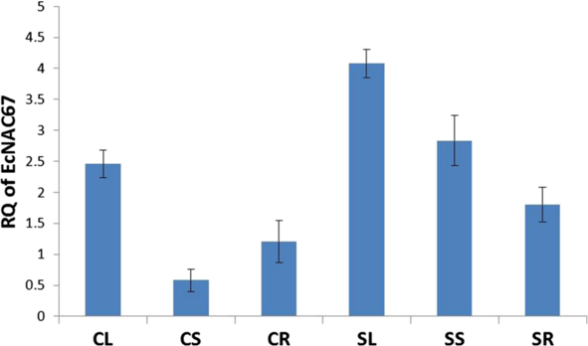
Expression pattern of *EcNAC67* transcripts in leaf, shoot and roots of finger millet plants grown under normal and salinity stress 300 mM of NaCl conditions. CL (Control Leaf); CS (Control Shoot); CR (Control Root); SL (Stress Leaf); SS (Stress Shoot) and SR (Stress Root). Error bars are the SE for three biological replicate

qRT-PCR analysis of salinity responsive expression pattern in control and salinity stressed leaves, shoots and roots of Trichy 1 revealed that, *EcNAC67* transcript was found to be abundant in leaves, followed by roots and minimum in shoots (Fig. 1). Transcript abundance was found to be highly up-regulated in all the three tissues during salinity (300 mM) and the level of up-regulation was found to be the highest in shoots followed by leaves and roots (Fig. 1).

### Cloning, sequencing and *in silico* characterization of *EcNAC67*

**Fig. 2:**
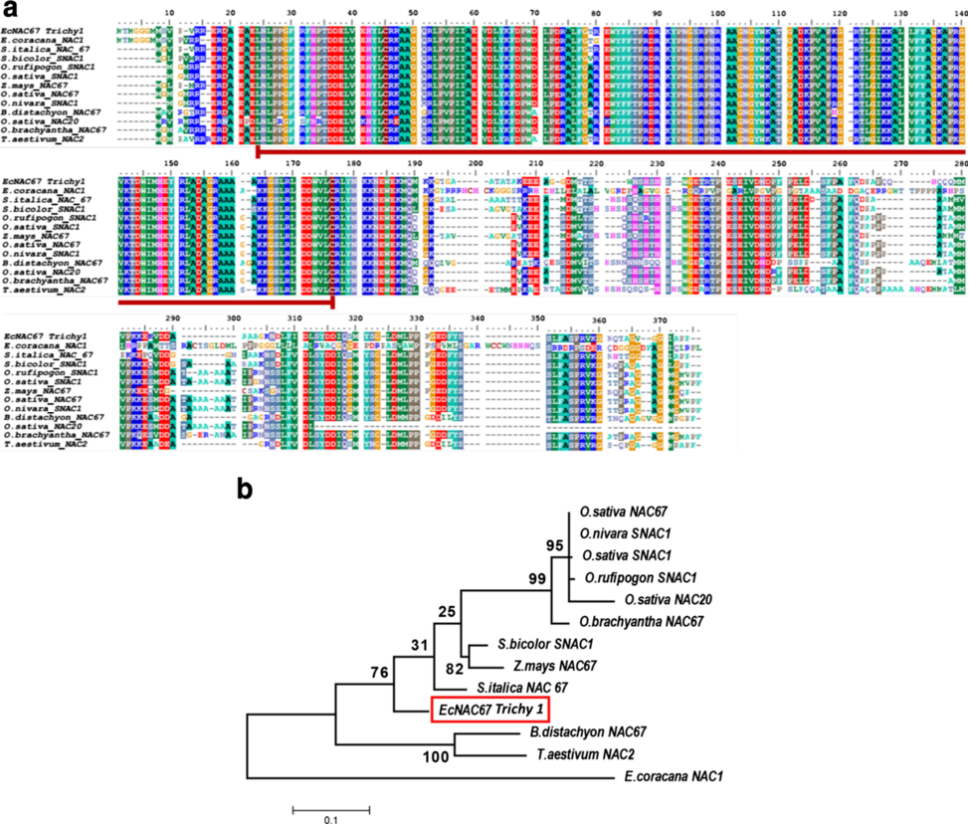
**a** Multiple sequence alignment of amino acid sequences of *EcNAC67* with NAC family members of selected plant species. Gaps (dashed lines) were introduced for optimal alignment. The region underlined with red bar indicates the conserved NAC-domain. Alignments were performed using ClustalW program. **b** Phylogenetic relationship between *EcNAC67* and NAC members of other plant species. The phylogenetic tree was constructed with the MEGA 6.0 software using Maximum Likelihood method, and the bootstrap values are in percent

Full length cDNA encoding putative NAC transcription factor was isolated from the salinity tolerant finger millet genotype Trichy 1 using gene specific primers designed based on the alignment of finger millet transcript contigs against rice homologue (LOC_Os03g60080) through RT-PCR. Cloning and sequence analysis revealed that it belongs to NAC 67 family (based on the structural domain) of transcription factors. *EcNAC67* shared significant homology with various NAC 67 family members viz., 87 % with *S. italica* (XP_004981295), 79 % with *O. brachyantha* (XP_006650786.1), 77 % with *Z. mays* (XP_008644394.1) and 75 % with *B. distachyon* (XP_003563852.1). It was predicted that it encodes for a protein of 322 amino acids with a molecular mass of 35.98 kDa and pI of 6.34. Conserved domain analysis revealed that *EcNAC67* contains two functional domains namely, N-terminal NAC domain (24–176 AA) which was found to be highly conserved (Fig. 2a) and a C-terminal domain which was exhibiting greater degree of variation across different members. Prediction of putative structure of *EcNAC67* protein revealed that *EcNAC67* contains 9 coils, 3 alpha helices and 5 beta-strands (see Additional file 2). Prediction of sub-cellular localization using ProtComp v9.0 online tool suggested that *EcNAC67* was a typical nucleus localization protein with a certainty score of 0.9 to1. Phylogenetic analysis of *EcNAC67* with other NAC family members of different species showed that *EcNAC67* formed a separate lineage from already reported *E. coracana* NAC as well as other NAC transcription factors reported from other species (Fig. 2b). Evolutionary divergence between sequences using Poisson correction model showed that *EcNAC67* was having least divergence with *S.italica* NAC67 and *S.bicolor* SNAC1 (see Additional file 3).

### Generation and characterization of transgenic rice plants over-expressing *EcNAC67*

**Fig. 3:**
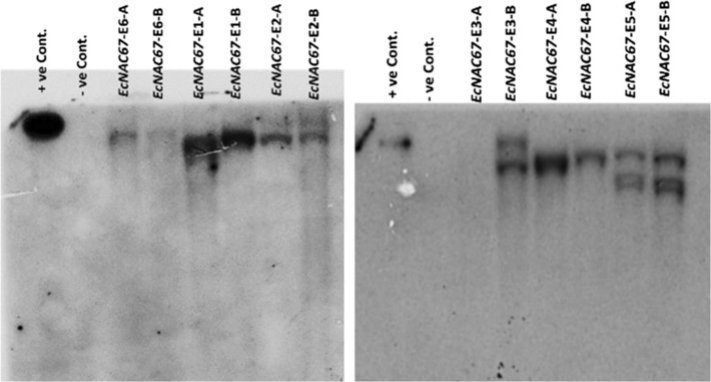
Southern hybridization analysis of *EcNAC*67transgenic rice (ASD16)linesin T_0_ generation; where + ve control is a linearized plasmid (pCAMBIA1300 + *ECNAC67*), −ve control is Non-transgenic ASD16 and *EcNAC67* (E1 to E6) are independent transgenic events; A and B represents two different transgenic plants of respective lines; About 20ug of genomic DNA was digested with *BamHI*and hybridized using Hygromycin (*hpt*) probe

Ninety two putative T_0_ transgenic plants were screened for transgene integration by PCR using primers specific to selectable marker gene hygromycin (*hpt*), transgene (*EcNAC67*) and also *vir* gene of *Agrobacterium*. All transgenic plants were found to be positive for *hpt* and transgene *EcNAC67* but were negative for *vir* gene of *Agrobacterium* showing that PCR amplification is not due to contaminating *Agrobacterium*. Two PCR positive transgenic plants (T_0_) were selected from each transgenic event and used for confirmation of transgene integration by Southern blot analysis. Results of southern blot analysis indicated that three transgenic lines namely, *EcNAC67*-E1, *EcNAC67*-E4 and *EcNAC67*-E6 were found to contain single-copy of the transgene; two transgenic lines namely, *EcNAC67*-E2 and *EcNAC67*-E3 were found to harbor two copies of the transgene and one transgenic line *EcNAC67*-E5 was found to possess 3 copies of the transgene (Fig. 3). One of the plants (Plant # A) from a putative transgenic line *EcNAC67*-E3 was found to be positive for the presence of transgene in the PCR analysis, but did not show hybridization signals in Southern blot analysis (Fig. 3). Southern confirmed and PCR positive transgenic lines were selfed and harvested individually.

Fifty seeds from each T_0_ lines were germinated in soil filled portrays and allowed for germination. PCR analysis using gene specific primers of both transgene and *hpt* revealed that 35–38 plants out of 50 plants were positive in single copy insertion lines (i.e. *EcNAC67*-E1 *EcNAC67*-E4 and *EcNAC67*-E6); 42 – 47 plants out of 50 were positive in two copy insertion lines (i.e., *EcNAC67*-E2 and *EcNAC67*-E3) showing a normal 3:1 Mendelian segregation ratio. PCR positive T_1_ plants were selfed and T_2_ transgenic lines were evaluated for their tolerance against abiotic stresses viz., drought and salinity.

### Over-expression of *EcNAC67* confers tolerance against salinity in rice

Non-transgenic and transgenic ASD16 lines were allowed to germinate in petri plates containing 75, 100 and 150 mM NaCl solutions and effect of salinity on the development of shoot and roots was measured on 10^th^ day. Salinity stress had significant effect on the growth of both shoot and root in non-transgenic ASD16 than the transgenic lines. All the transgenic rice lines were found to possess relatively less reduction in their shoot and root length when compared to non-transgenic ASD16 at 75 mM, 100 mM and 150 mM NaCl stress (Fig. 5). Non-transgenic ASD16 plants were found to have very small root at 150 mM NaCl stress but transgenic lines were having 3–4 times longer roots suggesting that the over-expression of *EcNAC67* in rice confers enhanced level of tolerance against salinity stress.

At 100 mM NaCl stress during vegetative stage, non-transgenic ASD16 plants exhibited growth retardation and wilting of terminal leaves at 11 days after stress at which all the transgenic lines were found to be healthy (Fig. 6a). At 33 days after stress, non-transgenic ASD16 plants were found to be severely affected and most of the leaves were found to be dried (Fig. 6b). All the transgenic lines retained greenness in leaves and had higher root/shoot biomass (Fig. 6d). Non-transgenic ASD 16 plants showed 60 % reduction in the total biomass during salinity stress where as in the transgenic lines it ranged between 31 – 44 % (Fig. 6d). After 33 days of 100 mM NaCl stress, all the plants were allowed for recovery by transferring to pots filled with soil and irrigated using normal water. All the transgenic lines were able to recover completely within 15 days after revival and were able to reach maturity and set seeds, whereas non-transgenic ASD16 plants were not able to revive from salinity injury and died (Fig. 6c and e).

### Over-expression of *EcNAC67* improves drought tolerance in rice

To understand the effect of *EcNAC67* over-expression on drought tolerance, T_2_ transgenic plants were grown in pots and severe drought stress was imposed. Both the non-transgenic and transgenic ASD 16 lines were subjected to approximately equal intensity of stress by allowing the soil moisture in pots to reach around 15 – 16 %. Upon drought, transgenic plants showed much delayed leaf-rolling symptom when compared to non-transgenic ASD16 plants (Fig. 7). All the transgenic plants were able to maintain 20 % (approx.) higher relative water content in the leaves (Fig. 8).

Upon re-watering, transgenic lines were able to revive and resumed growth rapidly when compared to non-transgenic plants (Fig. 7). Non-transgenic plants showed 47 % reduction in final plant height whereas transgenic lines showed height reduction between 15-21 % (Table 1). Further, reduction in grain yield/plant was 69.6 % in non-transgenic ASD 16 whereas reduction among transgenic lines ranged from 23.3 % to 29 % with exception to *EcNAC67*-E2 transgenic line where yield reduction was around 50 % (Table 1). Further, drought induced spikelet sterility was found to be higher in the non-transgenic ASD16 (40.82 %) than in the transgenic lines where it ranged between 11.70 and 21.84 % (Table 1).

**Table 1:** Agronomic performance of non-transgenic and transgenic ASD16 lines under normal and salinity stress conditions

	Plant height (cm)		Panicle length (cm)		Spikelet sterility %		Yield (g/plant)	
	Control	Stress	Control	Stress	Control	% reduction over control during salinity stress	Control	Stress
Non -transgenic ASD16	95.40 ± 0.95	50.23 ± 1.81	27.30 ± 0.52	18.55 ± 1.05	23.98 ± 0.81	40.82 ± 1.76	21.82 ± 0.68	6.64 ± 0.80
*EcNAC67*-E1	94.83 ± 1.02	80.23 ± 3.21	27.17 ± 0.14	20.33 ± 0.27	22.84 ± 0.93	11.70 ± 0.67	23.02 ± 1.40	16.29 ± 0.77
*EcNAC67*-E2	94.20 ± 0.99	74.05 ± 0.88	27.50 ± 1.06	19.25 ± 0.53	25.24 ± 0.87	21.84 ± 0.52	23.44 ± 0.65	11.65 ± 0.18
*EcNAC67*-E3	92.00 ± 1.27	78.43 ± 2.58	29.75 ± 0.88	23.57 ± 0.24	23.41 ± 0.97	15.76 ± 0.51	20.88 ± 0.14	14.41 ± 0.43
*EcNAC67*-E4	94.15 ± 1.17	75.43 ± 0.99	26.25 ± 0.88	21.67 ± 1.19	23.35 ± 0.04	20.88 ± 1.19	21.75 ± 2.15	16.49 ± 0.39
*EcNAC67*-E5	95.50 ± 1.41	75.43 ± 1.42	26.67 ± 0.72	19.83 ± 1.21	25.90 ± 1.30	20.79 ± 0.55	22.50 ± 0.46	17.26 ± 0.32

### Drought/salinity responsive expression pattern of transgene

**Fig. 4:**
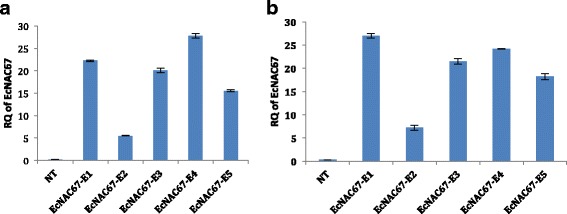
**a** Abiotic stress responsive expression pattern of transgene (*EcNAC67*) in Non-transgenic and transgenic ASD16 lines analyzed through qRT-PCR at 100 mM NaCl stress (NT:Non Transgenic, E1-E5: Transgenic lines). **b** Drought stress responsive expression pattern of transgene (*EcNAC67*) in Non-transgenic and transgenic ASD16 lines analyzed through qRT-PCR (NT:Non Transgenic, E1-E5: Transgenic lines)

qRT-PCR analysis of transgene expression in leaves of control, salinity and drought stressed plants of both non-transgenic and transgenic ASD16 showed that expression of transgene was absent in the non-transgenic ASD16 rice plants whereas all the transgenic lines were found to possess significantly increased level of transgene expression under stress conditions (Fig. 4). Among the transgenic lines, single copy lines (*EcNAC*67-E1 and *EcNAC*67-E4) were found to have maximum induction of transgene expression under both drought and salinity treatments when compared to the lines having multiple integrations of transgene (Fig. 4).

## Discussion

Drought and salinity are becoming major abiotic stresses limiting agricultural productivity worldwide. Predicted climate change is expected to increase the frequency of occurrence of these stresses and posing serious threat to global food security. Developing drought and salinity tolerant crop varieties will help in sustaining increased productivity under agricultural areas that are prone to such stresses. Conventional breeding efforts are resulting in a slow progress in achieving this goal due to complexity of mechanisms controlling tolerance against these stresses and lack of reliable high throughput phenotyping. In this context, use of biotechnological tools *viz.,* marker assisted breeding and genetic engineering offers us a powerful tool for genetic manipulation of these traits. Under genetic engineering, one of the promising strategies is to modulate the expression levels of stress responsive transcription factors that might regulate wide array of downstream genes/pathways and thus bringing the desired levels of tolerance to plants. Among the TFs, NAC transcription factors were shown to provide enhanced abiotic stress tolerance by regulating a wide array of stress related genes [27, 32, 47–49].

Recently, through various genome-wide sequencing and gene expression profiling experiments, several NAC TF family members have been identified and characterized in Arabidopsis, rice, wheat and other plants [50–57]. It has been demonstrated that over-expression of stress-responsive NAC TFs can significantly improve abiotic stress tolerance in plants [18, 26, 34, 35, 58, 59]. In this context, identification and characterization of novel stress responsive NAC TFs from resilient crop species like finger millet can provide greater insight into this unique group of transcription factors. In our previous study, a salinity responsive NAC67 transcription factor (homologous to rice NAC TF; LOC_Os03g60080) was identified through RNA-sequencing in a set of contrasting finger millet genotype differing for their degree of salinity tolerance [42]. In the present study, full length cDNA encoding stress inducible *NAC67* TF of finger millet was isolated, cloned and characterized. Results of this study confirmed that *EcNAC67* might act as a key TF in imparting abiotic stress tolerance.

Cloning, sequencing and analysis of deduced amino acids sequence of *EcNAC67* suggested that it shared significant similarity with already reported NAC67 family of monocots *i.e. S. italica, S. bicolor and Z. mays*. Phylogenetic analysis showed genetic relatedness of *EcNAC67* with NAC67 of *S. italica* (84 %) and *S. bicolor* SNAC1 (81.7 %). *EcNAC67* was found to possess highly conserved N-terminal DNA binding domain when compared with other stress responsive NAC TFs and highly variable C-terminal transcriptional activation domain.

Among several NAC TFs reported in rice, OsSNAC1 [18], SNAC2 [48] and Arabidopsis *RD26* [28], *ANAC019*, *ANAC055*, and *ANAC072* [60], very few were found to be stress-responsive. In this study, expression analysis of *EcNAC67* revealed its salinity stress responsiveness where *EcNAC67* transcripts were highly up-regulated under long term high salinity stress in leaves, roots and shoots of salinity tolerant finger millet genotype Trichy 1, indicating its involvement in salinity stress tolerance.

**Fig. 5:**
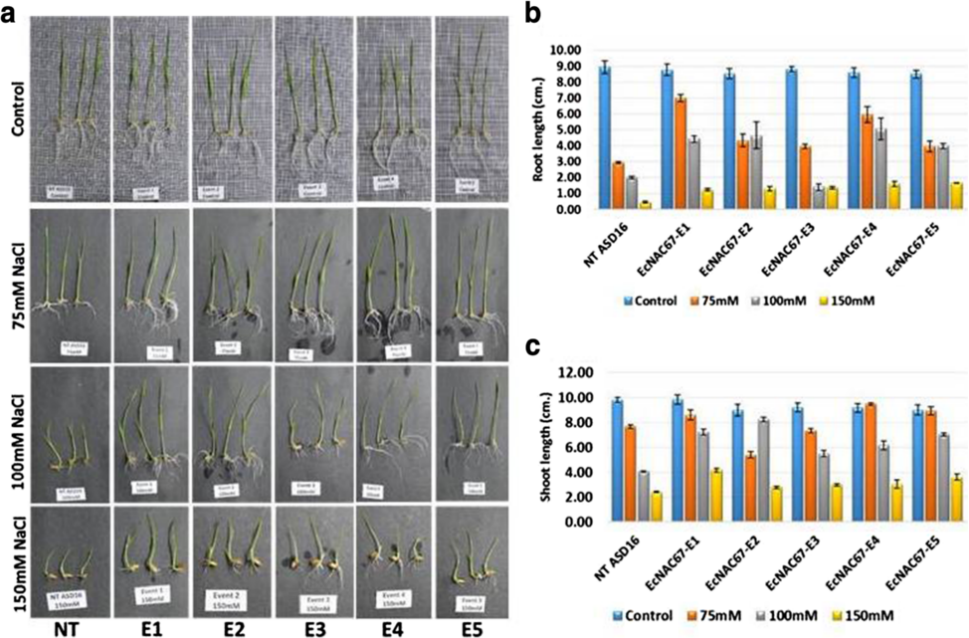
**a** Response of *EcNAC67* transgenic rice plants (E1-E5) and non-transgenic ASD16 (NT) under different levels of salinity stress during germination stage. **b** Root length measured in non-transgenic ASD16 (NT) and transgenic lines (E1-E5) on 10^th^ day after germination at different concentrations of NaCl. **c** Shoot length of non-transgenic ASD16 (NT) and transgenic lines (E1-E5) on 10^th^ day after germination at different concentrations of NaCl

Ultimate aim of this study was to validate the role of salinity responsive *EcNAC67* from finger millet for abiotic stress tolerance in rice. As there were several reports indicating that constitutive over-expression of transcription factors resulted in growth retardation in transgenic plants, *EcNAC67* was cloned under the control of a stress inducible (*RD29*) promoter and used for transforming an elite rice cultivar ASD16. Putative transgenic lines were identified through PCR analysis and confirmed through southern hybridization (Fig. 3). In T_1_ generation, positive plants were identified through PCR analysis and the results showed the segregation of transgene in 3:1, following typical Mendelian segregation pattern. PCR positive T_1_ transgenic lines were forwarded to T_2_ generation and evaluated for their tolerance against salinity at both germination and vegetative stage along with non-transgenic controls. At germination stage, progenies of transgenic ASD16 lines over-expressing *EcNAC67* showed better germination and shoot/root length under salinity stress (Fig. 5).

**Fig. 6:**
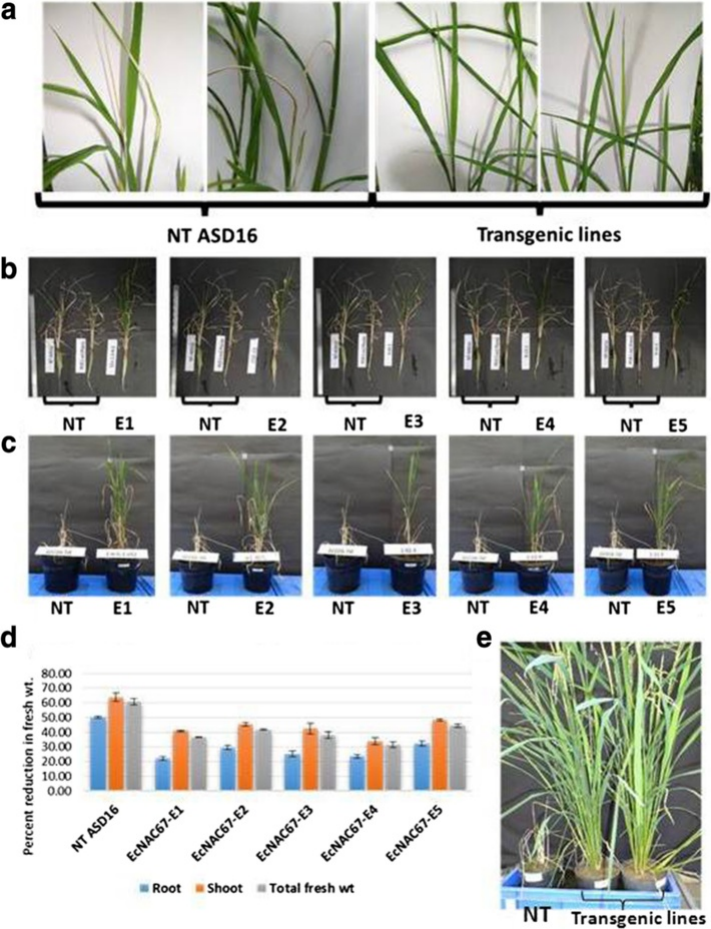
**a** Response of non-transgenic ASD16 plants and *EcNAC67* transgenic lines after 11 days of 100 mM NaCl stress during vegetative stage. **b** Response of *EcNAC67* transgenic lines (E1-E5) after 33 days of 100 mM NaCl stress during vegetative stage; where NT represents non transgenic ASD16. **c** Response of *EcNAC67* transgenic lines (E1-E5) after 15 days of revival from salinity stress. **d** Reduction in root length, shoot length and total plant biomass in response to salinity stress recorded in transgenic (E1-E5) and non-transgenic ASD16 plants (NT ASD16). **e** Phenotypic response of non-transgenic ASD16 (NT) and transgenic plants at maturity after relieving from salinity stress

Transgenic lines exhibited better growth rate and found to possess lesser number of dried leaves when compared to the non-transgenic plants during salinity. All the transgenic lines were found to exhibit enhanced level of salinity tolerance when compared to non-transgenic ASD16 plants by recording lesser reduction in their root, shoot and total biomass during salinity. Similar observations were made by Liu et al. [33], where transgenic cotton plants over-expressed with O*SNAC1* were found to accumulate more biomass when compared to the non-transgenic controls. There was no significant difference between the transgenic and non-transgenic ASD16 plants in their root length/growth under normal condition(s), but all the transgenic lines were found to possess significantly longer roots than non-transgenic ASD16 plants during salinity stress. Furthermore, all the transgenic ASD16 plants were found to recover rapidly from salinity induced injuries when compared to the non-transgenic plants when both were allowed to grow under normal conditions after 33 days of stress (Fig. 6c).

**Fig. 7:**
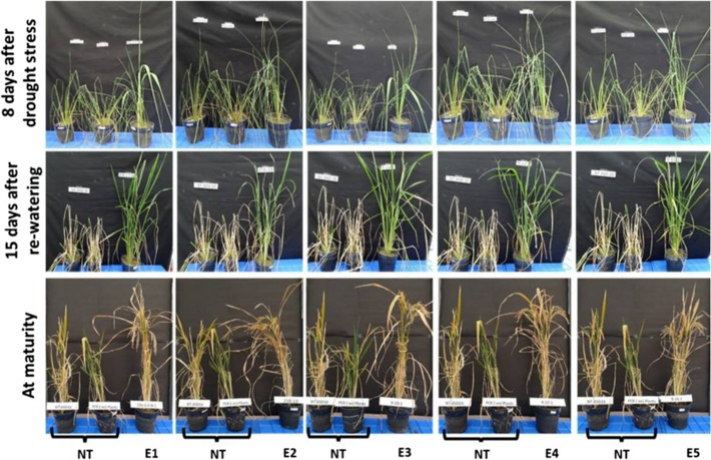
Phenotypic response of non-transgenic and transgenic ASD16 lines against drought stress; where “NT” is Non transgenic ASD16 and “E1-E5” are transgenic lines

**Fig. 8:**
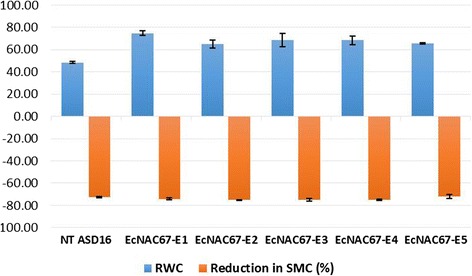
Relative water content in leaves of Non Transgenic (NT) and transgenic ASD16 lines when same level of drought stress was imposed in terms of soil moisture content

All the transgenic rice lines (T_2_) were evaluated for their drought tolerance ability in comparison with non-transgenic ASD16 plants. Non-transgenic ASD16 plants started showing drought induced leaf rolling and drying symptoms on 6^th^ day after stress whereas transgenic lines showed much delayed leaf-rolling symptoms (Fig. 7). RWC (%) is a widely used parameter to determine the plant’s internal water status and is a direct reflection of the ability of the genotypes to retain turgidity during water deficit conditions [61]. In this study, all the transgenic rice lines engineered with *EcNAC67* were found to retain 17-22 % higher RWC than the non-transgenic ASD16 plants at more or less equal intensity of drought (Fig. 8). This enhanced water retention capacity of *EcNAC*67 engineered transgenic rice lines might be due to better stomatal regulation during dehydration similar to the previous reports on *SNAC1* [18] and *TaNAC67* [35]. Further experiments are needed in this aspect to understand the precise role of *EcNAC67* in providing enhanced tolerance against drought stress. Further, all the transgenic lines were found to show better and rapid recovery upon rehydration when compared to non-transgenic ASD16 suggesting multiple roles of *EcNAC67* in protecting cellular proteins/enzymes/organelles during dehydration apart from regulation of water loss through stomata. Grain yield of both non-transgenic and transgenic ASD16 lines were found to be significantly affected by intermittent drought but the % reduction in yield was much higher in non-transgenic ASD16 than most of the transgenic lines. Lesser reduction in the grain yield of transgenic rice plants may be due to relatively lesser reduction in spikelet fertility (11.7 % to 21.84 %) than that of their non-transgenic counterpart where it showed around 40.8 % spikelet sterility. Drought stress negatively affects successful pollination and fertilization by decreasing the amount of viable pollen thus leading to spikelet sterility [62] indicating that non-transgenic ASD16 had undergone severe drought stress during panicle/pollen development as compared to transgenic lines at equal intensity of soil moisture stress.

The qRT-PCR analysis of the *EcNAC67 *transgene did not show any detectable levels under control condition as it’s was driven by a stress inducible promoter, *RD29* (Data not shown). qRT-PCR analysis of transgene expression in the transgenic lines revealed that two lines possessing single copy insertions (*EcNAC67*-E1 and *EcNAC67*-E4) were found to exhibit maximum level of expression under both salinity and drought stressed conditions (Fig. 4) as compared to the lines having multiple copies of transgene which may be attributed to co-suppression of transgene expression in case of multiple copy events as reported earlier [63]. Abundance of *ECNAC*67 transcripts was found to be highly correlated with root proliferation and development in the transgenic plants suggesting the probable role of *EcNAC67* in root development as reported in few other studies *viz., EcNAC*1 in tobacco [26], *AtNAC2* and *SNAC1* in rice [33, 47].

## Conclusions

Results of this study indicated that *EcNAC*67 can serve as a novel source for engineering salinity/drought tolerance in crop plants. Even though growth retardation is a common adverse physiological disturbance reported in transgenic plants over-expressing TFs, morphological and agronomical characters of *EcNAC67* engineered rice plants were comparable to non-transgenic ASD16 plants under normal well-watered conditions. No adverse effects were noticed in terms of plant height, panicle length and grain yield/plant. This shows the practical applicability of *EcNAC67* for genetic improvement of abiotic stress tolerance in rice and other agricultural crops of economic importance.

## Availability of data and materials

Nucleotide sequence of the reported candidate gene *EcNAC*67 from finger millet (*Eleusine coracana L.*) is available in the NCBI-GenBank database (Accession # KU500625). All other supporting data are included as additional files.

## Additional files

Additional file 1: Table showing the list of primers used in the study. (PDF 42 kb) Additional file 2: Secondary structure of deduced EcNAC67 protein sequence predicted by PSIPRED protein structure prediction server. (PDF 283 kb) Additional file 3: Table showing the estimates of Evolutionary Divergence among NAC homologs. Analyses were conducted using the Poisson correction model. All positions containing gaps and missing data were eliminated. There were a total of 241 positions in the final dataset. Evolutionary analyses were conducted in MEGA6. (PDF 191 kb)
