# Supplementary material for: Over-expression of a NAC 67 transcription factor from finger millet (Eleusine coracana L.) confers tolerance against salinity and drought stress in rice
Source: BMC Biotechnol. 2016 May 11;16(Suppl 1):35. doi: 10.1186/s12896-016-0261-1 (PMC4896240; doi:10.1186/s12896-016-0261-1)
Supplement: Additional file 1: — Table showing the list of primers used in the study. (PDF 42 kb) [file 12896_2016_261_MOESM1_ESM.pdf]

Additional file 1:- List of primers used in the study

**Primer Name**	**Sequence (5'-3')**
**Primer for isolating full length gene EcNAC67 and PCR analysis of transgenic lines**	
NAC -F	cgc***ggatcc***CAGGAGGGAGAGAGGAAAGAG
NAC -R	cgc***ggtacc***CGGATCAGGTTCAGGTTCTTCG
Hyg -F	TACACAGCCATCGGTCCAG
CaMV35S-R	ACCTCCTCGGATTCCATTGC
Vir-F	ATCGACGCCTCCAAAGTGAC
Vir-R	AAACGCGAGGAGAAGATTGA
Primer used for qRT PCR	
NAC RT-F	TCAGCAGCAGATGATGGTG
NAC RT-R	CGGATCAGGTTCAGGTTCTTCG
Ubiquitin -F	AGAAGCGCAAGAAGAAGACG
Ubiquitin -R	GCGTCGTCCACCTTGTAGA
Actin -F	GGCATCACACCTTCTACAAC
Actin -R	TCATCTTCTCACGGTTAGC
Primer used for isolating probe for Southern blot analysis	
Hpt S-F	GCTGTTATGCGGCCATTGTC
Hpt S-R	GACGTCTGTCGAGAAGTTTG
