# Supplementary material for: Over-expression of a NAC 67 transcription factor from finger millet (Eleusine coracana L.) confers tolerance against salinity and drought stress in rice
Source: BMC Biotechnol. 2016 May 11;16(Suppl 1):35. doi: 10.1186/s12896-016-0261-1 (PMC4896240; doi:10.1186/s12896-016-0261-1)
Supplement: Additional file 2: — Secondary structure of deduced EcNAC67 protein sequence predicted by PSIPRED protein structure prediction server. (PDF 283 kb) [file 12896_2016_261_MOESM2_ESM.pdf]

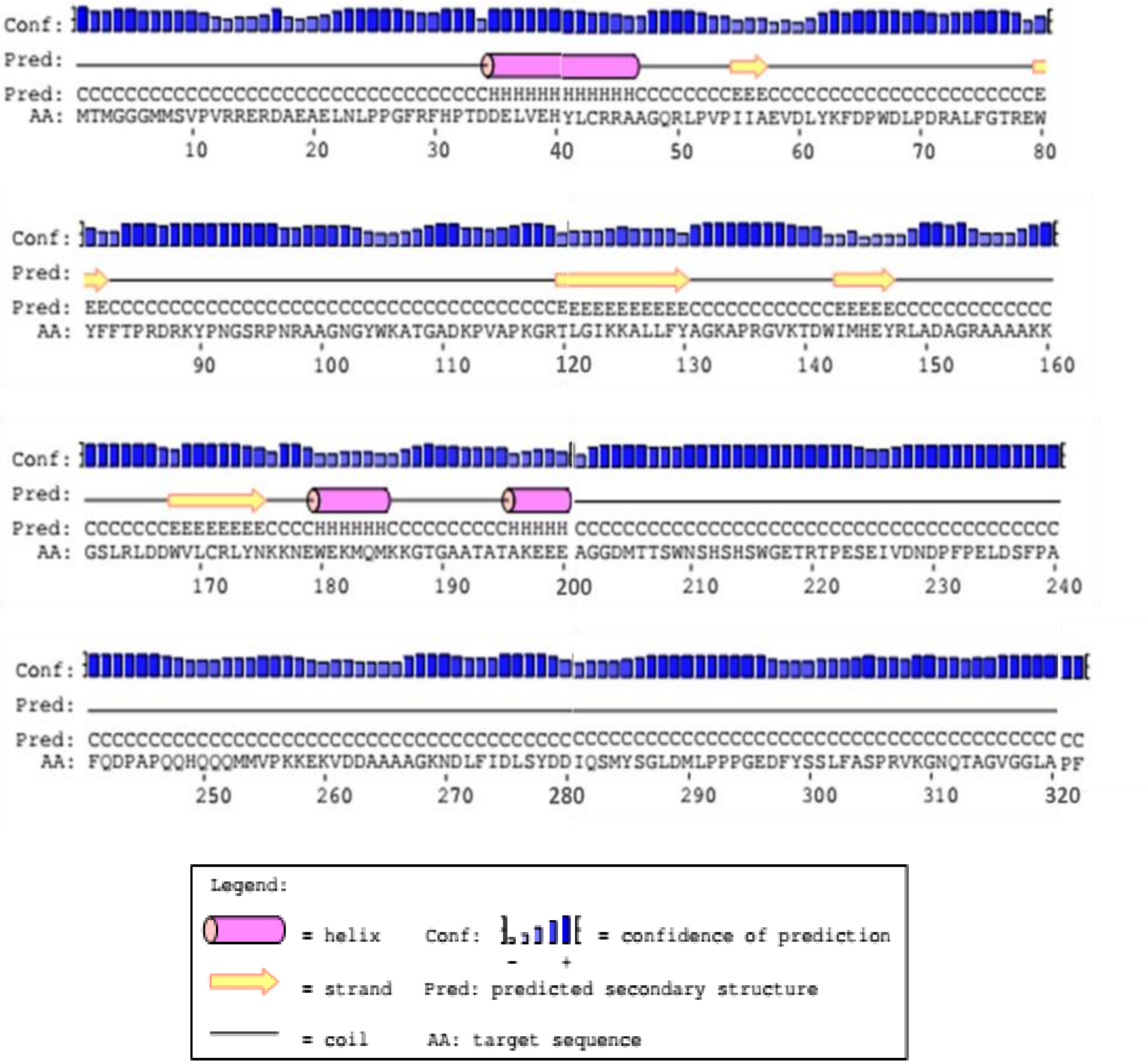

Additional file 2. Secondary structure of deduced EcNAC67 protein sequence predicted by PSIPRED protein structure prediction server
