# Supplementary material for: Over-expression of a NAC 67 transcription factor from finger millet (Eleusine coracana L.) confers tolerance against salinity and drought stress in rice
Source: BMC Biotechnol. 2016 May 11;16(Suppl 1):35. doi: 10.1186/s12896-016-0261-1 (PMC4896240; doi:10.1186/s12896-016-0261-1)
Supplement: Additional file 3: — Table showing the estimates of Evolutionary Divergence among NAC homologs. Analyses were conducted using the Poisson correction model. All positions containing gaps and missing data were eliminated. There were a total of 241 positions in the final dataset. Evolutionary analyses were conducted in MEGA6. (PDF 191 kb) [file 12896_2016_261_MOESM3_ESM.pdf]

Additional file 3. Estimates of Evolutionary Divergence between among NAC homologs. Analyses were conducted using the Poisson correction model. All positions containing gaps and missing data were eliminated. There were a total of 241 positions in the final dataset. Evolutionary analyses were conducted in MEGA6 .

	EcNAC67 Trichy1	***E. coracana NAC1***	***S. italica NAC67***	***S. bicolor SNAC1***	***O. rufipogon SNAC1***	***O.sativa SNAC1***	***Z.mays NAC67***	***O.sativa NAC67***	***O.nivara SNAC1***	***B. distachyon NAC67***	***O.sativa NAC20***	***O. brachyantha NAC67***
E.coracana NAC1	0.297											
S.italica NAC67	0.096	0.303										
S.bicolor SNAC1	0.091	0.326	0.047									
O. rufipogon SNAC1	0.124	0.314	0.100	0.082								
O.sativa SNAC1	0.119	0.314	0.096	0.078	0.008							
Z.mays NAC67	0.114	0.320	0.064	0.042	0.100	0.096						
O.sativa NAC67	0.119	0.314	0.096	0.078	0.008	0.000	0.096					
O.nivara SNAC1	0.119	0.314	0.096	0.078	0.008	0.000	0.096	0.000				
B.distachyon NAC67	0.177	0.320	0.207	0.197	0.202	0.197	0.222	0.197	0.197			
O.sativa NAC20	0.162	0.373	0.142	0.128	0.064	0.055	0.147	0.055	0.055	0.243		
O. brachyantha NAC67	0.119	0.320	0.091	0.078	0.021	0.013	0.091	0.013	0.013	0.202	0.064	
T.aestivum NAC2	0.181	0.320	0.197	0.177	0.177	0.172	0.186	0.172	0.172	0.119	0.233	0.177
